# Proteomic Analysis of Grape Berry Cell Cultures Reveals that Developmentally Regulated Ripening Related Processes Can Be Studied Using Cultured Cells

**DOI:** 10.1371/journal.pone.0014708

**Published:** 2011-02-17

**Authors:** Ramaschandra G. Sharathchandra, Charmaine Stander, Dan Jacobson, Bongani Ndimba, Melané A. Vivier

**Affiliations:** 1 Department of Viticulture and Oenology, Institute for Wine Biotechnology, Stellenbosch University, Stellenbosch, South Africa; 2 Proteomics Research Laboratory, Department of Biotechnology, University of Western Cape, Bellville, South Africa; Cairo University, Egypt

## Abstract

**Background:**

This work describes a proteomics profiling method, optimized and applied to berry cell suspensions to evaluate organ-specific cultures as a platform to study grape berry ripening. Variations in berry ripening within a cluster(s) on a vine and in a vineyard are a major impediment towards complete understanding of the functional processes that control ripening, specifically when a characterized and homogenous sample is required. Berry cell suspensions could overcome some of these problems, but their suitability as a model system for berry development and ripening needs to be established first.

**Methodology/Principal Findings:**

In this study we report on the proteomic evaluation of the cytosolic proteins obtained from synchronized cell suspension cultures that were established from callus lines originating from green, véraison and ripe *Vitis vinifera* berry explants. The proteins were separated using liquid phase IEF in a Microrotofor cell and SDS PAGE. This method proved superior to gel-based 2DE. Principal component analysis confirmed that biological and technical repeats grouped tightly and importantly, showed that the proteomes of berry cultures originating from the different growth/ripening stages were distinct. A total of twenty six common bands were selected after band matching between different growth stages and twenty two of these bands were positively identified. Thirty two % of the identified proteins are currently annotated as hypothetical. The differential expression profile of the identified proteins, when compared with published literature on grape berry ripening, suggested common trends in terms of relative abundance in the different developmental stages between real berries and cell suspensions.

**Conclusions:**

The advantages of having suspension cultures that accurately mimic specific developmental stages are profound and could significantly contribute to the study of the intricate regulatory and signaling networks responsible for berry development and ripening.

## Introduction

Grapes are among the most widely cultivated fruit crops in the world. Grape berries like other non-climacteric fruits undergo a complex set of dynamic, physical, physiological and biochemical changes during development, which can be divided into three major phases. The double sigmoidal pattern of berry development begins with a first growth phase characterized by berry size increases due to frequent cell division and subsequent cell expansion. The initial growth stage is followed by a short lag phase characterized by a lack of cell expansion and low biochemical activity. The end of the lag phase is termed véraison and is an important stage of molecular and metabolic switching [1], [2]. The second period of sigmoidal growth follows; in this ripening phase acid levels decrease and sugars begin to accumulate while the berry softens and produces ripening pigments and aroma compounds [3].

Recently a number of investigations have been carried out to understand the complex changes in gene and protein expression, as well as metabolite profiles that occur during berry development. Results obtained using ESTs (expressed sequence tags) [4]; Affymetrix *Vitis* Gene-Chips [5], [6], metabolite analysis [7], two dimensional gel electrophoresis [8], [9], [10], [11] and isobaric tags for relative and absolute quantification (iTRAQ) [12] demonstrated dynamic changes in the transcriptome and proteome during berry ripening.

The investigations mentioned have either harvested berries at single time points or throughout berry development. Variations between berries within a cluster and the general vineyard variation due to various environmental factors is a major impediment towards ensuring synchronous populations of berries. Common practice is to obtain berries of the same size, but it is known that asynchrony in growth between berries distorts the growth curves obtained from mixed populations [3]. Studies have shown a spread of 10–23 days between the softening dates of different clusters on a vine and also in a vineyard [2]. In addition, a study by [6] using morphological and global transcriptional profiling indicated that individual berries of the same size follow distinct developmental timing patterns within the cluster, not just at the morphological level, but also at the molecular level. This aspect is critical when considering the application of systems biology tools to the study of grapevine berries, since these technologies rely on characterized, homogenous and representative samples.

Plant cell cultures are an attractive alternative source to a whole plant for various physiological and biochemical studies. Plant cells in a culture are independent of geographic, seasonal variations and varying environmental factors. They offer a defined production system, which ensures uniform quality and rapid yield. In addition, plant cells can be manipulated to perform stereo and regio-specific biotransformation for production of desirable compounds [13]. Grapevine cell suspensions have been used previously to demonstrate the role of various phenylpropanoid metabolites against *Botrytis cinerea* elicitors [14], to produce bioactive stilbenes [15], study glucose regulation of monosaccharide transport [16], sugar sensing and calmodulin requirements [17] and the implication of signaling pathways in elicitor-induced defense responses [18]. Grapevine cell suspension cultures have also been used to study individual berry-specific processes. Hiratsuka et al. [19] studied the effects of abscisic acid (ABA), sugar and anthocyanin formation. The trends on ABA and titratable acid accumulation were similar to the berries grown on vines during ripening.

Berry cultures have not yet been specifically tested for their suitability to study the dynamics of berry development and ripening. To this end we developed organ-specific, synchronized cell suspension cultures from biochemically and developmentally characterized berry explants harvested from the green, véraison and ripe stages. Here we report on the development of a proteomic workflow to evaluate the use of berry cell suspension cultures as a tool to study grape berry ripening. Two-dimensional polyacrylamide gel electrophoresis (2D–PAGE), consisting of isoelectric focusing (IEF), using immobilized pH gradient (IPG) gels in the first dimension and sodium dodecyl sulphate polyacrylamide gel electrophoresis (SDS–PAGE) in the second dimension, was compared with a method where a Microrotofor was used for IEF in free solution of the berry cytosolic proteome. The latter method improved the recovery of proteins. Protein identification, statistical analysis of the data and comparison with known data on berry proteins confirmed that the cytosolic proteins isolated from the different berry cultures showed differential expression of berry proteins which corresponded to the patterns observed in berries ripening on a vine. Moreover, signaling and lower abundance proteins, not previously detected in berries, were identified from the cultures.

## Results

### Characterization of berry explants and establishment of callus cultures and suspensions

**Figure 1:**
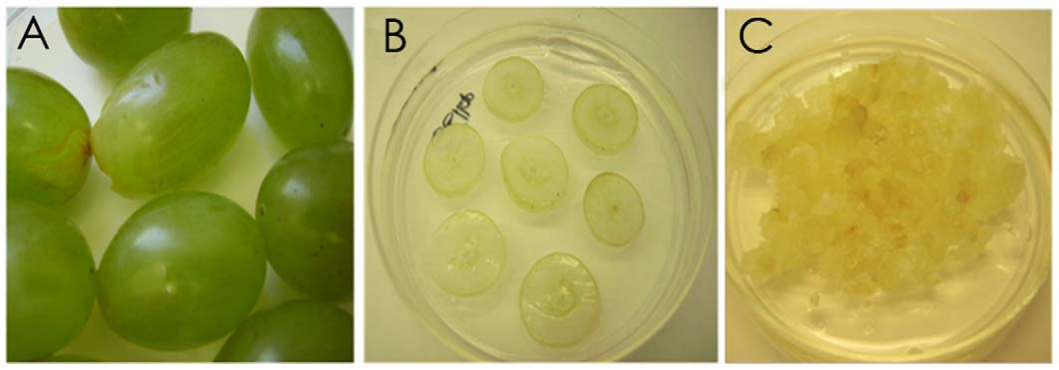
Development of berry-derived callus cultures. A. Dauphine berries as explants at the ripe stage; B. Berry explants on callus-initiation medium; C. Somatic callus cultures originating from the plated berry explants. These cultures were used to established suspension cultures.

**Figure 2:**
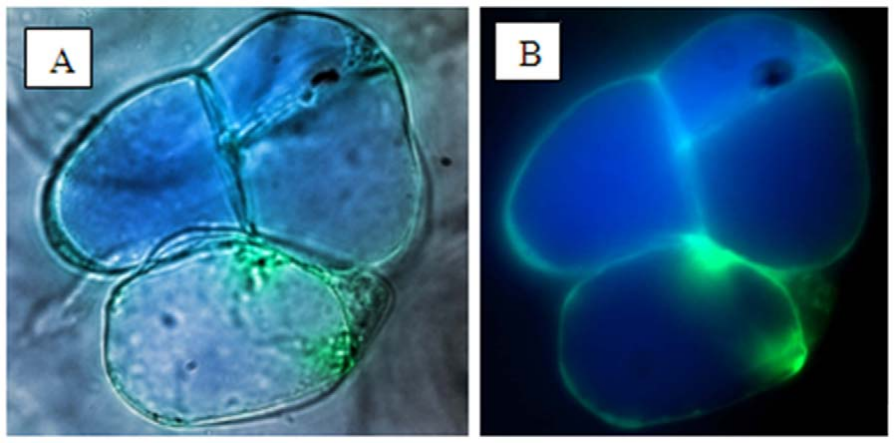
Dauphine berry suspension culture cells observed under light microscopy (A) and stained with fluorescence staining markers (B); Green: vacuolar membrane marker and blue: vacuolar lumen marker. Life imaging microscope: magnification of 40X.

**Figure 3:**
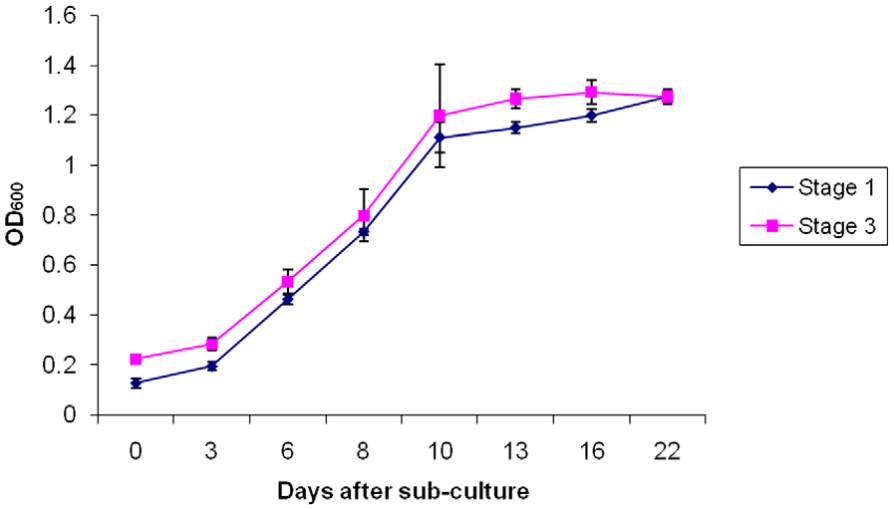
Growth curve analysis of suspension cells derived from berry explants. Shown are growth curves recorded for stage I (from green berries) and III (from ripe berries) suspension cultures that were monitored over a 21 day period. Samples for proteomic evaluation were taken at 7 (log phase) and 14 days (early stationary phase) after sub-culture.

The berry explants spanned the major stages of berry development and ripening (as reported [20]) and exhibited the typical double sigmoidal growth curve characteristic of berry development. The phenotypical berry characteristics at each of the sampling points are described in Table 1. Somatic callus cultures were successfully initiated from all nine sampling points, but explants in the green and ripe stages callused more readily than the samples in the lag and véraison stage of berry development (Table 1). Non-embryogenic callus developed from the skin and pulp sections of the explants (berry slices) within three to six weeks of culturing. The callus was translucent to cream in colour, loose and watery with a shiny appearance (Figure 1). The suspension cultures were successfully initiated with these callus clumps and consisted of friable, fast dividing callus cells that dispersed homogenously into the culture medium. The suspension cultures consisted of actively growing, homogenous, thin walled and highly vacuolated cells (Figure 2, and Supporting Information S1). The suspension cultures exhibited a typical sigmoidal growth curve with a lag-phase (0–2 days), log-phase (3–10 days) and a stationary phase (11–21 days) (Figure 3). The cultures could be maintained by weekly sub-culturing to provide actively proliferating mid-log phase cells for initiating fresh cell suspension cultures.

**Table 1:** Sampling, phenotypical description and callus production from *Vitis vinifera* cv. Dauphine berries.

Sampling number	Phenological stage (indicated as weeks post anthesis)	General description of *Vitis vinifera* cv. Dauphine berries	Callus production#	Suspension cultures subjected to proteomics
1	3	Small, hard and green with small soft seeds	152/158 (96%)	
2	5 ½*	Pea size, hard and green with soft seeds; berry closure	171/195 (87%)	Suspension designated Stage I(originating from green berries)
3	7	Green and hard, increasing in size; seeds hardening and becoming brown and difficult to cut	123/158 (77%)	
4	9	Green, hard with volume growth slacking off; seeds are hard and brown	52/114 (45%)	
5	10 ½*	No volume growth; some berries start to soften and change colour	77/143 (54%)	Suspension designated Stage II(originating from véraison berries)
6	11	Berries starts to soften and increase size and yellow pigments	45/144 (31%)	
7	12 ½	Colour development occurs, berries increase in size; berries soften	101/142 (71%)	
8	15*	Berries soft and sweet and increasing in size and ripe colour	98/142 (94%)	Suspension designated Stage III(originating from ripe berries)
9	19	Berries fully ripe	126/140 (90%)	

# Explants showing callus production/total explants cultures (percentage).

*Callus lines from these explants were used to established suspension cultures I-III for proteomic analysis.

### Protein recovery

The extraction method and the two dimensional separation system, using free flow electrophoresis and SDS-PAGE led to better separation and focusing of the soluble proteins from the grapevine suspension cultured cells when compared to the normal IPG based 2DE previously attempted (comparisons presented in Supporting Information S1). Moreover, the latter method was not effective to resolve the soluble proteins from the véraison-stage explants. We therefore used the Microrotofor, an apparatus for IEF in free solution to perform a proteomic comparison of the Stage I-III suspension cultures, instead of IEF based on IPG gels. The TCA/acetone-based method used for protein extraction yielded reproducible amounts of proteins from all the suspensions (Table 2), but it was clear that protein yields from Stages I-III differed. Stage I cells (originating from green berry explants) yielded up to 0.83 mg protein per gram fresh weight of cells, whereas protein yields decreased to 0.57 mg. g^−1^ of cells in stage II cultures (originating from véraison berries). The protein concentration again increased in stage III cultures (suspension cells derived from callus generated from explants of ripe berries) to reach 0.79 mg. g^−1^ of cells. Regulated vacuum harvesting by Microrotofor cell allowed for high levels of protein recovery (77–91%; Table 2).

**Table 2:** Soluble protein recovered from grape berry suspension cultures.

Ripening stage of berry explants	Growth phase of suspension cultures	Protein concentration (mg) per gram of tissue after extraction^#^	Protein recovery (%) after separation in Microrotofor cell
Green (Stage I)	Early Log	0.827±0.02	86.25±0.48
	Late Log	0.833±0.003	87.13±0.06
Véraison (Stage II)	Early Log	0.565±0.005	77.19±0.20
	Late Log	0.572±0.004	77.37±0.42
Ripe (Stage III)	Early Log	0.799±0.003	90.26±0.12
	Late Log	0.797±0.003	90.77±0.13

Extraction performed with a slightly modified method of Garavaglia et al. (2010a).

### Expression profiles and identification of proteins

The gel image analysis showed that twenty six distinct and well resolved bands (densitometry values higher than 0.1% of the total intensity of the gel) were common to all the ripening stages. In order to identify unique proteins expressing in different ripening stages, gels were analyzed for bands with lower densitometry values, but not common to all the ripening stages. However, very few such bands were recorded and they were not distinct enough to be separated into individual Gaussian units. Therefore, such bands were not included for relative quantification analysis and identification. Post normalization, protein bands were relatively quantitated based on densitometry values and are listed in Table S1 (also see Table S2 for the densitometric values for the identified bands reported in Table S1). All the protein bands displayed dynamic changes in their expression profiles in stages one, two and three. A comparison on protein expression trends in the present study with previous reports on transcript or protein expression in grape berries has been provided in Table S3.

**Figure 4:**
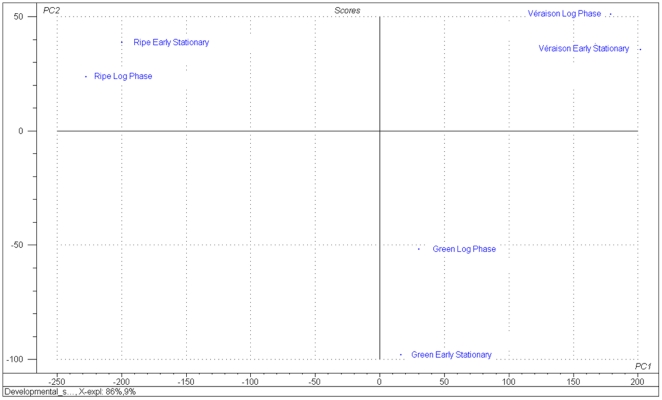
Principal component analysis as performed on a matrix consisting of the objects as defined by the log and early stationary phase cultures derived from different growth stages and the variables defined as the average expressed values of each protein (band) detected in each of those cell cultures. Ripe, véraison and green refer to the different ripening stages of berry suspension cells (cultures I-III, respectively) used in the study. Log and early stationary refer to the growth phase of the cells sampled for the study.

Principal component analysis (PCA) was performed on a matrix consisting of the objects as defined by the early and late log phase cell cultures derived from each stage of berry development and the variables defined as the average expressed values of each protein (band) detected in each of those cell cultures. The early and late log phase matrices of stages I-III plot quite closely (Figure 4), confirming the homology in the samples per developmental stage. The PCA results show that the total proteome signal from the different stages, when considered as individual matrices, cluster far apart from each other, confirming that the berry cultures originating from berry explants in different developmental stages, maintained significant differences in the cultured state. Given the tight groupings in the two sampling points (log and early stationary phase), only the data from the log phase will be reported on in all further sections.

**Figure 5:**
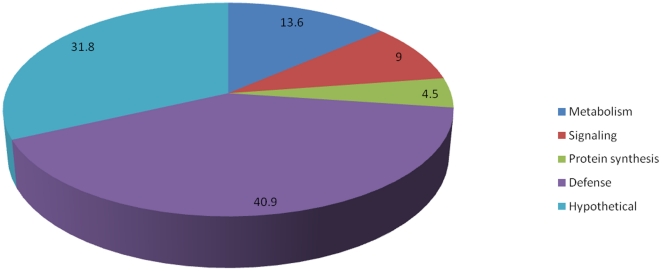
Functional categories of the identified proteins and their distribution indicated in percentage.

Among the 26 bands subjected to MALDI-TOF/MS analysis, 22 bands were identified. A protein was considered to be positively identified if a minimum of four peptides matched, showed ≥10% sequence coverage and a Molecular Weight Search (MOWSE) score of ≥70 (p<0.05). The detailed list of the identified proteins with their theoretical molecular weights, pIs, number of matched peptides and their respective expression profiles are reported in Table S1. The identified proteins could be categorized into different functional classes including metabolism, signaling, protein synthesis and defense (Figure 5).

Since translated open reading frames (ORFs) from grapevine genome projects are available in public databases, the searches identified many non-described and hypothetical polypeptides [14]. Among the 22 protein bands identified, seven proteins were either hypothetical or unnamed protein products. A large group of seven bands were identified as Class I β-1, 3-glucanase from *V. vinifera*. Five among the seven β-1, 3-glucanases were encoded by gi|82547239. The other two were encoded by gi|22431926 and gi|1658001. With the use of Aldente, many of the proteins identified in our study could be matched to grapevine-specific proteins, as well as to isoforms (Table S4) previously generated using iTRAQ and a custom built tryptic peptide database [12].

## Discussion

Grape berry ripening is an important process determining the quality of the harvested crop. Transcriptomic and metabolomic analyses of *V. vinifera* cv. Cabernet Sauvignon have revealed a dynamic change in regulatory networks controlling ripening [7]. Proteomic studies with 2DE and MALDI-TOF [9], iTRAQ and a custom generated tryptic peptide database [12] on wine grape cultivars have also contributed valuable data regarding ripening. Gribaldi et al. [9] Reported identification of 118 differentially regulated spots in a proteomic evaluation of seven different ripening stages of *V. vinifera* cv. Nebbiola Lampia. However, only 25% of the identified proteins could be matched to *V. vinifera* due to the limited availability of annotated genomic information. In addition, differential expression profiles could not be unambiguously assessed for many spots because several proteins were present in individual spots. Lucker et al. [12] reported an improvement in the sensitivity and accurate quantification with their method in which four different ripening stages of *V. vinifera* cv. Cabernet Sauvignon were used. Consistency in ratiometric data remained limited and variance in detecting proteins in biological replicates was problematic and attributed to seasonal changes.

In addition to the difficulty of working with a woody perennial that is influenced by seasonal changes, berries on a bunch vary significantly within a specific season. Lack of synchrony in berry ripening is a result of functional autonomy of individual berries and studies on the accumulation of monoterpene glycosides, compound import and global transcriptomic profiling using individual berries [6] have shown that individual berries in a bunch function independently of each other. This poses a challenge for molecular biology-based systems biology approaches in general and proteomics in particular. Therefore, the use of organ-specific suspension cultures to study a developmentally regulated process such as ripening, is an interesting experimental goal with potentially wide ranging impact.

In the present study we used a table grape cultivar, Dauphine, to establish synchronous cell suspensions from berries harvested at different ripening stages. Somatic cell cultures (on plates) were successfully established from berry explants in all stages of berry development and ripening, although the second phase of berry development (the lag phase ending in véraison) yielded less explants capable of developing callus (Table 1). Through successive transfers, synchronized callus cultures were developed from green, véraison and ripe berries. Given the fact that explants (typically differentiated and mature tissues/organs) de-differentiate and form meristematic cells during the process of callus formation, it is assumed that most of the “context” of the original explants is lost. In this system, PCA analysis confirmed that the total cytosolic proteome of the cultures originating from the different explants still remained significantly different after the callus initiation stages and development of synchronized suspension cultures (Figure 4). Furthermore, the PCA of proteome dynamics employed in this study strongly indicated the existence of a tight homology in sampling and a firm partitioning between the different ripening stages. The results clearly indicated that the variances between ripening stages I, II and III dominated over the protein quantities of the log and early stationary phase sampling.

### Advantages of the Liquid-phase IEF based proteomics system

Despite the recognized importance of proteomics to complement other systems biology approaches that are being applied to grapevine, several technical difficulties with the methods available remain important constraints. Liquid chromatography-based mass spectrometry has common problems with reproducibility due to technical limitations [21]. Other grapevine proteomic studies have focused on sub-fractionated proteomes such as membranes [22] and grape skins during ripening to overcome some problems [8]. Liquid phase IEF using Microrotofor as a strategy in 2DE has been recently well reported in literature. It has been used for proteomic analysis including bone marrow tissues [23], *Leishmania amazonensis* culture [24], rat liver [25], *Escherichia coli* cells [26], mice serum [27], *Leptosphaeria maculans* and *L. bicolor* [28]. The advantages of this method include separation of a wide survey of proteins in both acidic and basic narrow pI ranges onto which pooled fractions were successfully resolved by SDS PAGE. To our knowledge, liquid-phase IEF had never been used to resolve plant proteomes. This technique improved the recovery of proteins from berry cell cultures, as well as enhanced their subsequent 2DE separation (see comparative data in Supporting Information S1).

Our results showed that total protein concentrations dropped at véraison, compared to the protein levels extracted from cultures that originated from the green and ripe berry stages (Table 2). A similar pattern was observed in real berries where the concentration of soluble proteins decreased around véraison, with an increase up to five fold during ripening, but a strong reduction in the diversity of the polypeptides present [29].

2DE has some obvious technical disadvantages like the loss of low-abundance proteins, such as regulatory proteins, receptors, and proteins with key roles in cellular processes. Besides low-abundance proteins, other groups of proteins with specific properties like very small and very large proteins, alkaline proteins, and hydrophobic proteins are difficult to analyze by 2DE [30]. Fractionation by liquid-phase IEF is particularly beneficial for those proteins that are insoluble or otherwise do not separate well in other, gel-based IEF media. IEF in solution enables fractionation of proteins in their native state by isoelectric point (pI) because ampholytes are used to generate the pH gradient used for separation. Liquid-phase IEF efficiently displaces charged material toward both ends of the pH gradient (i.e. electrode chambers) thereby improving protein focusing [31].

As the Microrotofor Cell generated discrete fractions according to protein pIs, it enabled the use of a variety of pH gradients in this study to optimize protein separation conditions. Consequently, major proteins were likely to be prone to isoelectric precipitation and became less prominent, thus providing the ability to identify minor proteins. Although some proteins might have been irreversibly lost in the process, many more bands appeared and overall resolution improved. This method also allowed the analysis of all three berry suspension cultures, whereas the IPG-based 2DE system could not resolve the stage II cultures in our experiments.

### Protein identification

The differential gel analysis yielded 26 bands common to all the ripening stages (Table 2). 22 of the 26 bands were identified. Seven among the identified proteins was hypothetical, or had an unknown protein product. Previous grapevine proteomic experiments have also revealed high levels of hypothetical proteins [9], [10], [11], [32], which is probably linked to the fact that the grapevine genome is still poorly annotated due to the lack of adequate *in silico* comparisons. Grapevine genome annotation is an ongoing process and the next critical challenge is to adapt existing interpretive tools of model species to the specificities of the grapevine genome. Having grapevine-specific tools will increase the power and speed of proteomic and systems biology data analysis. By incorporating grapevine-specific ESTs and genome-derived proteomes, we were able to show that some of the proteins identified in our study (Table S3) matched several isoforms which had been previously reported by [12].

The protein identification focused only on common proteins of which differential expression patterns could be observed and were compared to known results from literature on grape berry ripening, or expression databases. Although some typical berry proteins that are known to have roles in berry development and ripening were identified, some expected proteins with known function and activity in the soluble cytoplasmic fractions were not present in our list of identified proteins. As an example, proteins such as invertases catalyze hydrolysis of sucrose, provided by the phloem conducting complex, into glucose and fructose during berry ripening. Invertase isoforms are either acidic or neutral and are localized in the cell wall, cytoplasm and vacuole. Based on protein motif analysis 10 to 12 putative acidic invertases are found in the grape genome sequence [33]. Most of the acidic invertases are targeted to the vacuole of the berry cells and the soluble activity can be measured provided appropriate extraction buffers are used. The extraction system used in our study promotes solubility of neutral and basic proteins as evident in the pIs of the identified proteins. Berry suspension cells have been previously used as a model to study sugar transport and regulation [34], but a more detailed investigation using modified extraction systems would be necessary to enrich for other proteins including invertases with different properties in a model cell suspension culture. In addition, sub-cellular fractionation of the cell walls would also be essential in order to understand the role of cell wall invertases as they constitute 4% of the total invertase activity [35].

The proportion of proteins involved in stress responses was higher in our list of identified proteins, if compared to the functional distributions previously observed in the proteome of whole berries [9] and isolated mesocarp tissues [36] in which these proteins ranged from 8% to 19% of the identified spots. Our results however paralleled a recent large-scale mRNA expression analysis on the three main berry tissues [32], as well as the skin proteome analysis of cultivar Cabernet Sauvignon where most of the proteins over-expressed at maturity were involved in pathogen response [8].

### Proteins identified in V. vinifera

Cytoplasmic malate dehydrogenase (MDH) identified in our study is the principal enzyme responsible for malate metabolism. Malate is actively synthesized and accumulates until véraison in the grape berry, while it becomes a preferential substrate for respiration at the onset of ripening. The cytoplasmic malate dehydrogenase (MDH) is thought to be responsible for malate catabolism and the mitochondrial isoform to be involved in malate synthesis [37]. Cytosolic NADP-dependent malic enzyme (ME) activity, which converts malate into pyruvate and CO_2_, is already high before véraison and is unlikely to control malate breakdown [38]. In our study the MDH expression is low at stage I, increases at stage II and is slightly higher in stage III. This corresponds with previously reported enzyme assays and proteomic expression profiles (Table S3).

Levels of glutamate dehydrogenase (GDH) from *V. vinifera* identified in our study were very high at stage I, decreased sharply in stage II and remained constant thereafter. The role of GDH in plants is still unclear; it is thought to play a role in glutamate degradation and metabolic recycling of ammonia, but it could also have an accessory role in ammonia assimilation. In grape berries, GDH activity peaks before véraison and the protein expression data confirmed the trend seen by [9]. Although the function of GDH in the cytosol is still unknown, it is possible that when it is induced in this cellular compartment, it plays an important role in the recycling of carbon and nitrogen molecules in source organs and/or at specific stages of plant development. GDH protein was also visualized in multivesicular bodies present in the cytosol of the senescing flower receptacle. The appearance of GDH in the cytosol at late stages of flower development was concomitant with the induction of both the enzyme activity and the amount of protein prior to the formation of the berries [39].

The most abundant class of proteins identified in our study is devoted to defense. Among the defense proteins Class I β-1, 3-glucanase comprises the biggest group. Seven identified protein isoforms were encoded by three different genes. All the identified β-1, 3-glucanases were similar in terms of expression profiles. These proteins recorded low expression at stage I, increased levels at stage II with a slight decrease during ripening. β-1, 3-glucanases belong to a group of proteins called pathogenesis-related (PR) proteins. They are synthesized in healthy grape berries in a developmentally dependent manner as a normal part of the ripening process, with véraison apparently being the trigger for PR gene expression [40]. In addition, to the development-dependent synthesis and accumulation of PR proteins, these proteins can also be induced in leaves and pre-véraison berries as part of an induced defense response against wounding, chemical elicitors, pathogen attack, or abiotic stress. The majority of the β-1, 3 glucanases have a role in response to microbial pathogens and developmental responses. β-1, 3 glucanases are present in multiple structural isoforms that differ in size, pI, primary structure, cellular localization and pattern of regulation [41], [42]. In other proteomic studies on grape berry ripening β-1, 3-glucanase demonstrated differential expression profiles but was mostly found to decrease or was undetectable during ripening (Table S3).

Ras-related GTP-binding protein identified in this study has a signaling role. The Ras super family comprises a large group of structurally and functionally conserved small GTP-binding proteins that function as two-state molecular ‘switches’ in numerous signaling pathways. Small GTP-binding proteins regulate diverse intracellular pathways, and include receptor-mediated signaling. The small GTP-binding proteins are activated by GTP binding, and inactivated by hydrolysis of GTP to GDP. They coordinate numerous downstream processes in plants such as hormone responses, cell growth and differentiation, pathogen defense and other abiotic stress responses [43]. In other plant species, it is involved in the phytochrome-mediated signal transduction as an auxin signal transducer in stems and seedlings. These proteins are further involved in protein transport, probably in vesicular traffic and eventually in the prenylation of proteins [44]. From our data, the expression is higher at stage I followed by a decrease at stage II and a slight increase later in stage III. In grape berries they have been reported to be involved in protein transport and prenylation with expression peaks at ripening, which conforms to our study (Table S3).

### Proteins identified in other plants

Several proteins identified in the present study belonged to different plant species. Ferredoxin NADP^+^ oxidoreductase (*Triticum aestivum*) identified in the present study is mainly metabolic in its function. Ferredoxin NADP+ oxidoreductase (FNR, EC 1.18.1.2) is a ubiquitous enzyme belonging to a large family of flavoproteins found in higher plants, eukaryotic algae, photosynthetic bacteria, and animals. This protein records a constant lower expression at green and véraison stages with a sharp increase at the ripening stage. In plants, this enzyme exists in two distinct forms as a photosynthetic (pFNR) and as a heterotrophic (hFNR) form. These two isoforms are encoded by different genes, linked to different metabolic pathways and are regulated by different factors [45]. Traditionally, pFNR has been considered to catalyze the final step in the photosynthetic electron transport chain. By contrast, a role for hFNR has been characterized for supplying reducing power in non-photosynthetic tissue to a variety of metabolic processes including nitrate assimilation. Multiple FNR forms are regulated independently and can interact and provide added flexibility to allow the plant to adapt to a changing metabolic environment [46]. Ferredoxin-NADP+ oxidoreductase (FNR) mediates the final step of photosynthetic electron flow by transferring electrons from ferredoxin to NADP+. The reducing power (NADPH) is used for a number of different reactions, such as carbon fixation, nitrogen metabolism, and lipid and chlorophyll biosynthesis, as well as for stromal redox regulation [47]. Electrons may also be abstracted directly from ferredoxin for the reduction of distinct components that play a role in the reducing reactions mentioned above [48]. Grapevines and berries have a complex system for nitrogen mobilization and partitioning. Since grape berries are only photosynthetically active for the period before véraison, hFNR may also actively work in controlling nitrate metabolism. However, its exact role in grape berries has to be clearly elucidated.

Ribosomal S2 protein identified in the current study was found to decrease in concentration in stage II and increased in stage III. Ribosomal S2 proteins take part in ribosomal biogenesis and protein synthesis. It has been shown to belong to a family that includes 40S ribosomal subunit 40 kDa proteins, putative laminin-binding proteins, NAB-1 protein and a 29.3 kDa protein from *Haloarcula marismortui*. The laminin proteins are predicted to be the eukaryotic homologue of the eubacterial S2 ribosomal proteins [49] and are said to have unrelated activities such as cell adhesion and ribosomal biogenesis. In berries during ripening these proteins might be involved in enhanced protein synthesis as it has been reported that protein concentration increases during complete maturation of the berries [29].

Our study also identified proteins involved in signaling responses. Kinases with ankyrin repeats (*Zea mays*) were identified for the first time in grape berry ripening related processes. However, this particular protein was found to match an ATP-dependent clp protease in the grapevine proteome. In our study the protein was expressed lowly at stage I, increased during stage II and showed a modest decrease in stage III. Other signaling proteins identified with a role in defense were the TIR NBS TIR protein and a LysM domain-containing receptor-like kinase (LysM RLK). In our study, the TIR NBS TIR protein had lower activities at both stage I and II with a sharp increase in stage III. To date, five principal classes of Resistance-genes have been identified, based upon conserved protein domains. The most abundant class is the cytoplasmic nucleotide-binding site-leucine-rich repeat (NBS-LRR) proteins. The NBS domain is important for ATP binding and hydrolysis and is believed to be involved in signal transduction, triggered by the presence of the pathogen. The TIR NBS subfamily of R proteins display homology between the N-terminal amino acid motif and the receptor domain in *Drosophila Toll* and basal mammalian Interleukin (IL) 1 immunity factors in animals [50].

A LysM domain-containing receptor-like kinase (LysM RLKs) was also identified for the first time during grape berry ripening. The expression of this protein dropped at véraison but increased again during ripening. LysM receptor kinases are involved in symbiotic signaling and defence against fungi. LysM domains are found in a variety of peptidoglycan and chitin binding proteins. In the plant kingdom, LysM domains are found in receptors of chitooligosaccharide and related compounds. It has been reported that these receptors are involved in the interaction between plants and microbes. Chitin is involved in inducing defence responses in both monocots and dicots [51]. Recently it has been reported that *Arabidopsis* CERK1 a receptor-like kinase with three LysMs also plays a key role in fungal perception by the plant [52]. It has been demonstrated that CEBiP (A LysM motif-containing plasma membrane kinase) directly binds to chitin oligosaccharides and plays an essential role in the perception of chitin and the induction of immunity. CEBiP of *Oryza sativa* contains two LysMs and is a glycoprotein with high affinity for chitin oligosaccharides. Knock-down of CEBiP resulted in suppression of chitin-induced defence response [53].

The proteins identified in non *V. vinifera* plant species need to be evaluated further in berries using molecular and bioinformatic tools but the berry cultures might provide excellent systems to study these proteins and their regulation.

In conclusion, the results presented in this study using berry suspension cells, combined with the liquid-phase IEF based proteomic experiments and multivariate data analysis show that berry cell cultures are suitable to study grape berry ripening. The fact that berry cultures derived from berry explants in different stages of development yielded protein expression patterns that could be matched to expression profiles found in berries provide an excellent platform for further studies, also on the metabolite level. Our results indicate that this type of suspension cultures could be used to model a range of protein activities found in developing berries. The advantages of having suspension cultures that accurately mimic specific developmental stages are profound and could significantly contribute to the study of the intricate regulatory and signaling networks responsible for berry development and ripening. We were able to identify proteins with a wide range of theoretical pI and molecular weights mainly due to improvements in the first dimension focusing with microrotofor and the expression trends for some of the identified proteins matched with those in literature. Further, to obtain a tissue specific understanding of ripening processes in the berry cell cultures it is important to delve deeper by further sub-cellular fractionation focusing on the cell wall and the secretome. Nonetheless, the results presented here establish that organ-specific berry cell cultures can be developed from all stages of berry development and that these cultures characteristically differ from each other. The observed proteins and their expression patterns that could be matched to patterns previously seen in berries provide further motivation to use these cultures to study berry development and ripening-related aspects of grapevine.

## Materials and Methods

### Plant material

*Vitis vinifer*a L. cv. Dauphine grape berries were sampled during the 2007–2008 growing season from the Irene farm in the Paarl valley, South Africa. Berries were collected at nine time points during the berry development season (spanning the period three to 19 weeks post anthesis), with 10–14 day intervals. For each time point, berries from three bunches were collected and their diameters recorded. Harvested berries were randomly divided into two batches. The one batch was immediately flash-frozen in liquid nitrogen and the other was kept on ice for callus initiation purposes. The frozen material was further processed by removing the seeds, while keeping the material frozen, and homogenized in an IKA A11 basic analytical mill (Sigma-Aldrich Z341789CH). The frozen tissue was subsequently analyzed for sugar, organic acid and pigment (chlorophyll and carotenoid) content (as reported in [20]) to characterize the berry development and ripening stages of the nine different sampling points.

### Establishing somatic callus lines from the different berry samples

Somatic callus lines were established as described by [54]. Harvested berries were washed in distilled water, and then agitated in a 7% (w/v) Calcium hypo-chloride solution for 15 minutes [55]. Subsequently the berries were rinsed three times in distilled water and de-seeded. The berries were sliced thinly (1–3 mm) and placed on solidified media containing 0.2 mg. l^−1^ kinetin and 0.1 mg. l^−1^ α-naphthalene acetic acid (NAA), 2% (w/v) sucrose, 250 mg. l^−1^ casein hydrolysate [54]. The medium was solidified with 3 g. l^−1^ phytagel (Sigma cat # P8169) and the pH was adjusted to 5.9. The numbers of berry explants developing callus were recorded and compared. The cultures were maintained at 25°C in the dark and were sub-cultured by transferring three to four grams of the developing callus masses onto fresh media every four weeks. To synchronize the cultures on the plate, sub-culturing and selection of similar callus continued until homogenous and prolific callus cultures were obtained.

Cell suspensions were initiated from callus cultures that originated from berry explants harvested at six, 11 and 15 weeks post-anthesis according to the method described by [54]. These time points corresponded to green, véraison and ripe berries and the suspensions were designated Stage I to III respectively. Four to five grams of sub-cultured callus, was transferred to a 250 ml Erlenmeyer flask, containing 100 ml of the callus initiation medium (without phytagel) and incubated on a rotary shaker (110 rpm) at 25°C in the dark. Cell suspensions were maintained by transferring 20 ml of a seven day-old culture into 80 ml of fresh medium. After three successive rounds of sub-culturing the cell suspensions were homogenous and synchronized. The growth curves of the cell lines were determined by measuring the OD_600_ of samples of the suspension cultures over a 21 day period. Three biological repeats and three technical repeats of each biological repeat were used. Somatic cells developing in the suspension cultures were also microscopically viewed, using an Olympus Cell® system attached to an IX-81 inverted fluorescence microscope equipped with a F-view-II cooled CCD camera (Soft Imaging Systems). From the suspension cultures, cells in the log phase and the early stationary phases were harvested for proteomic evaluation.

### Protein extraction from suspension cells

Cytocolic proteins were extracted according to a method of [56], with modifications. In brief, suspension cells generated from green, véraison and ripe berries (designated stages I-III) in their log and early stationary phases were separated from the growth medium using Mira cloth and ground in liquid nitrogen. Three grams (g) of each growth stage was used for extraction and three independent extractions were carried out for each growth stage. The cells were suspended in 10 ml of 12.5% (w/v) trichloroacetic acid (TCA) in 80% (v/v) acetone containing 2% (w/v) dithiothreitol (DTT) and 8 mM phenylmethanesulphonylfluoride (PMSF). The solution was left in a vortex shaker for approximately two hours and subsequently stored at −20°C overnight. Protein precipitate was separated by centrifugation (10, 000 rpm) at 4°C for 20 minutes. The protein pellet was washed thrice in ice cold acetone containing 2% DTT and 8 mM PMSF. The protein pellet was dissolved in 3 ml urea buffer containing 7 M urea, 2 M thiourea, 5 mM DTT, 4% (w/v) CHAPS (3*-*(3-Cholamidopropyl)dimethylammonio)-1-Propanesulfonic Acid), and 2% (v/v) Pharmalyte, pH 3–10 and constantly shaken for approximately 4 hours.

### Protein separation in the first dimension using free flow in-solution electrophoresis

The liquid phase IEF in the Microrotofor cell was conducted according to [31]. The protein samples (3 ml) were injected into the focusing chamber of the Microrotofor cell (Bio-Rad Laboratories Inc, Hercules, CA) using a 3 ml syringe until all ten compartments were equally loaded. The ion-exchange membranes, separating the electrode reservoirs and the focusing chamber, were equilibrated overnight in respectively 0.1 M H_3_PO_4_ (for a cation-exchange membrane) and 0.1 M NaOH (for an anion-exchange membrane). Both the loading and collection apertures on the opposite sides of the focusing compartments were sealed with adhesive tape. The focusing assembly was positioned in the cooling block and gently rocked with the use of a oscillating motor. The separation was conducted according to the manufacturer's instructions at a constant power of 1 W at 20°C. The separation typically occurred for 2.5 hours and was terminated 25 minutes after the current stopped decreasing.

Protein fractions from each compartment (200 µl) were collected with the use of a vacuum and the pH of the individual fractions was measured with a precision digital pH meter. Equal volumes (200 µl) of 20% (w/v) TCA were added to each of the fractions. Protein precipitates were collected by centrifugation at 10, 000 rpm for 10 min at room temperature. 500 µl of 80% (v/v) cold acetone was added to the precipitates, with subsequent vortexing, and centrifugation at 10, 000 rpm for 10 min at 2°C, after which the supernatant was carefully removed. This washing step was repeated twice to remove remaining TCA in the precipitates. These were air-dried and dissolved in urea buffer. The protein content of the total soluble protein was estimated by a modified Bradford assay using bovine serum albumin (BSA) as a standard [56].

### Second dimension separation and imaging

The separated fractions were further fractionated in the 2^nd^ dimension by SDS-PAGE. All gels were run in triplicate. Each individual fraction was loaded from left-right in a SDS gel, with the farthest left lane containing the most acidic-fraction. 50 µg of protein was loaded into each lane and the run was conducted at 70 V for 30 minutes, followed by 100 V for 2 hours. The gels were stained overnight in modified Coomassie Blue stain, destained and imaged using a Molecular Imager Pharos FX plus system (Bio-Rad Laboratories Inc., Hercules, CA). Digitized images at 100 µm resolution were analyzed using Quantity One analysis software (Bio-Rad Laboratories Inc., Hercules, CA). The gels corresponding to the different ripening stages were compared in order to attribute common band identities for the same band in each lane from different gel images. Before band detection and matching, images were aligned in relation to the gel set as a reference. Band detection and matching was carried out using the band matching tool, but was refined by manual band editing where needed. Protein bands were modeled as Gaussian distributions and their relative densitometry values (band densitometry values over the total densitometry values) and the relative front of the band was used for band matching. Relative quantitative analysis was performed on bands which were resolved well enough to be considered as two separate Gaussian units.

### Quantification and expression profiling

Densitometry values of the individual bands obtained from each replicate obtained were normalized against total densitometry signal (i.e. total protein content) present on the gel for each of the three repeats from three independent extractions. Values from replicates were averaged and the standard deviation for each band calculated. T-tests were done comparing each band derived from green, véraison or ripe cell cultures to determine if the differences seen were statistically significant. Principal component analysis was performed on a matrix consisting of the objects as defined by the early and late log phase cell cultures derived from each stage of berry development and the variables defined as the average expressed values of each protein (band) detected in each of those cell cultures. Developmental differential protein profiles were created by the log2 transform of each band value divided by the value obtained from the tissue cultures derived from green berries. All numeric calculations and transformations of the data were done via custom built perl programmes.

### Identification of proteins using Matrix-assisted laser desorption/ionization time-of-flight (MALDI-TOF) mass spectrometry (MS)

Bands of interest were identified by MALDI-TOF/MS using the protocol of [57] with slight modifications. Briefly, bands were excised manually and transferred into sterile micro centrifuge tubes. The gel pieces were washed twice with 50 mM ammonium bicarbonate for 5 min each and a third wash for 30 min, with occasional vortexing. The gel pieces were then destained twice with 50% (v/v) 50 mM ammonium bicarbonate and 50% (v/v) acetonitrile for 30 min, vortexing occasionally. The gel pieces were dehydrated with 100 µL acetonitrile for 5 min, and then completely dessicated using the Speed Vac SC100 (ThermoSavant, Waltham, MA). Proteins were in-gel digested with approximately 120 ng sequencing grade modified trypsin (Promega, Madison, WI) dissolved overnight in 25 mM ammonium bicarbonate at 37°C. The protein digestion was terminated by adding 50–100 µL of 1% (v/v) trifluoroacetic acid (TFA) and incubating the mixture 2–4 h at room temperature before storing the samples at 4°C until further analysis. Prior to identification, the samples were cleaned-up by reverse-phase chromatography using ZipTipC18™ columns (Millipore, Billerica, MA) pre-equilibrated in 100% (v/v) acetonitrile and then in 0.1% (v/v) TFA and eluted out with 50% (v/v) acetonitrile. One microlitre from each sample was mixed with the same volume of α-cynahydroxy-cinnamic acid (CHCA) matrix and spotted onto a MALDI target plate for analysis using a MALDI-TOF mass spectrometer (Voyager DE Pro Biospectrometry workstation (Applied Biosystems, Forster City, CA) to generate a peptide mass fingerprint. All MALDI spectra were calibrated using the sequazyme calibration mixture II (Applied Biosystems, Forster City, CA) containing angiotensin I, ACTH (1–17 clip, 1296.6853 daltons (Da)), ACTH (18–39 clip, 2093. 0867 Da), ACTH (7–38 clip 2465.1989 Da) and bovine insulin (3657.9294 Da). This instrument has a 337 nm Nitrogen Laser and is operated in the positive ion reflectron mode at 20 kV accelerating voltage. 0.5 mL of peptide extract was used for the MALDI-TOF/MS analysis and co-crystallized in the matrix a-cyano-4-hydroxycinnamic acid.

Monoisotopic peptide masses were assigned and used for databases research. The NCBI and MSDB peptide mass databases were searched using MASCOT Matrix Science, London, UK http://www.matrixscience.com/search_form_select.html). The searches were carried out against plant species. One missed cleavage per peptide was allowed, a mass tolerance of 100 ppm was used and some variable modifications were taken into account, such as carbamidomethylation for cysteine and oxidation for methionine. The identification of proteins was based on a positive result using MASCOT's “Probability Based MOWSE Score”. In addition, grapevine-specific proteome tryptic digest databases were created from both the translation of coding regions found within the *Vitis vinifera* genome sequence [58], as well as the EST-derived protein set utilized by [12]. Aldente [59] was used to compare the mass peptide fingerprints generated by MALDI-TOF/MS to these grapevine-specific proteomes.

## Supporting Information

Supporting Information S1 Macro-and microscopic view of the suspension cultures (Figure S1); IPG-based 2DE of total soluble proteins from suspension cultures I (green) and III (ripe) (Figure S2); and SDS PAGE of total soluble proteins from suspensions I-III representing different ripening stages of the explants and separated by liquid phase IEF in a microrotofor cell (Figure S3).
(2.14 MB DOC)

Table S1 MALDI-TOF identification of proteins after band matching to compare their relative expression profiles.
(0.08 MB DOC)

Table S2 The densitometry values for each of the individually identified proteins supports the expression levels reported in Table S1, as well as the PCA analysis in Figure 4.
(0.03 MB XLS)

Table S3 Proteomic studies on grape berry ripening and expression profile comparison of proteins identified in our study with the published literature.
(0.07 MB DOC)

Table S4 Further identification of proteins in grapevine-specific databases.
(0.02 MB XLS)
