# Supplementary material for: Proteomic Analysis of Grape Berry Cell Cultures Reveals that Developmentally Regulated Ripening Related Processes Can Be Studied Using Cultured Cells
Source: PLoS One. 2011 Feb 17;6(2):e14708. doi: 10.1371/journal.pone.0014708 (PMC3040747; doi:10.1371/journal.pone.0014708)
Supplement: Supporting Information S1 — Macro-and microscopic view of the suspension cultures (Figure S1); IPG-based 2DE of total soluble proteins from suspension cultures I (green) and III (ripe) (Figure S2); and SDS PAGE of total soluble proteins from suspensions I-III representing different ripening stages of the explants and separated by liquid phase IEF in a microrotofor cell (Figure S3). (2.14 MB DOC) [file pone.0014708.s001.doc]

**Supporting document 1**

**Characteristics of suspension cultures**

**
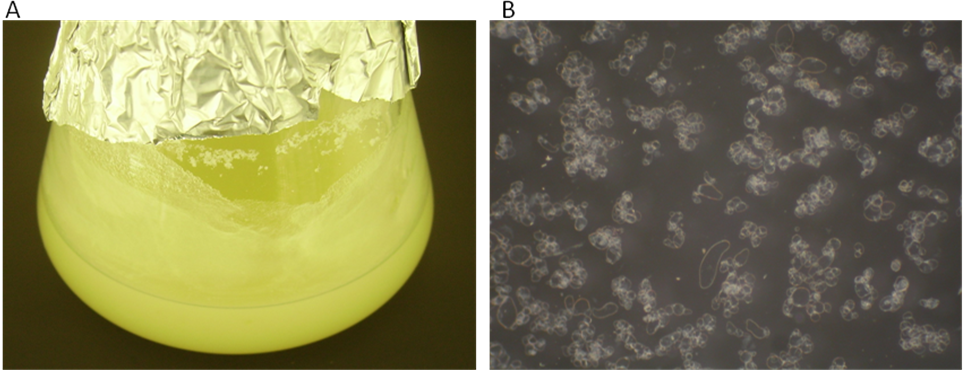
**

**Supplementary Figure 1.** Macro- and microscopic view of the cell suspensions. (A) Photograph of a cell line after 7 days of growth. (B) A sample of the cells from a 7-day old suspension culture viewed under a light microscope. The cells are actively dividing, thin walled and highly vacuolated.

**IPG based 2-DE Gel Electrophoresis of total soluble proteins isolated from suspension cultures originating from green, véraison and ripe berries**

Soluble protein samples (150 μg) were mixed with 0.8% (w/v) dithiothreitol (DTT), 0.2% (v/v) ampholytes pH 3-10 (BIO-RAD, Hercules, CA), 0.002% bromophenol blue and the volume was adjusted to 125 μL using urea buffer. The samples were then used to passively rehydrate linear 7 cm IPG strips, pH range 4-7 (BIO-RAD) overnight at room temperature. The strips were subjected to isoelectric focusing (IEF) using the Ettan™ IPGphor II™ (GE Healthcare, Amersham, UK), in a step-wise programme for a total of 10, 000 volt hours at 20°C. Prior to evaluation in the second dimension, the strips were equilibrated twice for 10 min with gentle shaking in an equilibration buffer (6 M urea, 2% (w/v) SDS, 0.05 M Tris-HCl, pH 8.8 and 20% (v/v) glycerol), firstly containing 1% (w/v) DTT and then 2.5% (w/v) iodoacetamide. The strips were then loaded to 12% SDS-PAGE gels and electrophoresed at 120 V until the bromophenol blue dye reached the bottom of the gel plates (about 90 min). The gels were stained with Coomassie Brilliant Blue, imaged with the PharosFX™ plus molecular imager scanner (BIO-RAD).


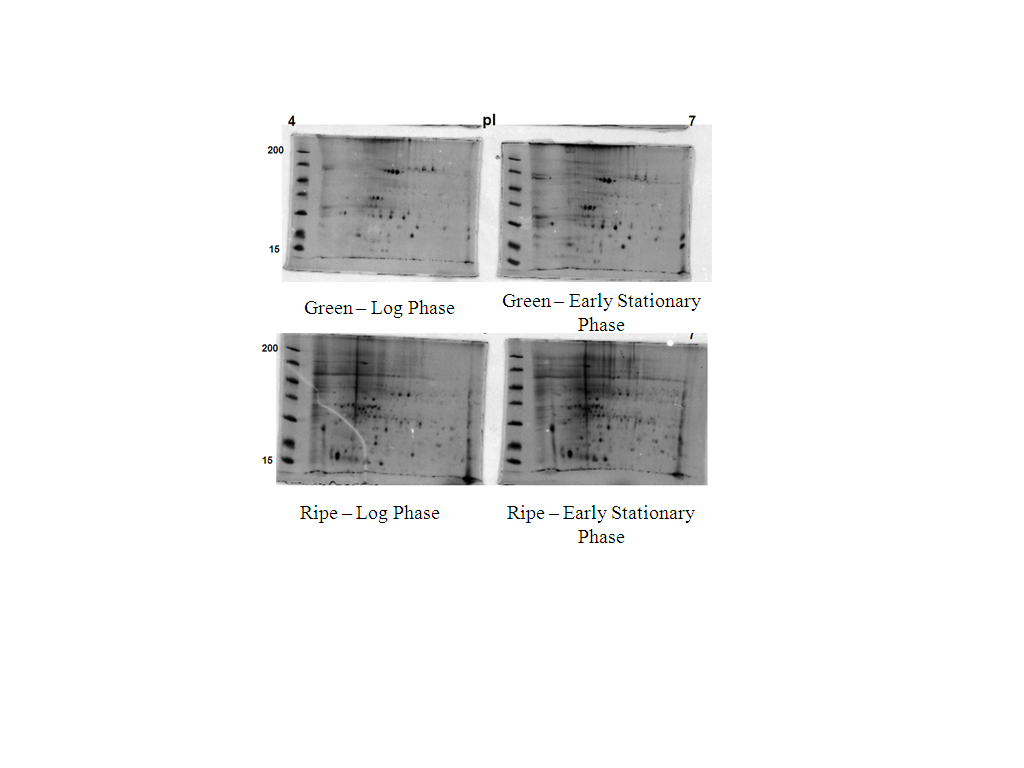


**Supplementary Figure 2**. IPG-based 2DE of total soluble proteins from suspensions cultures I (green) and III (ripe). Soluble proteins from suspension cultures II (véraison stage explants) could not be resolved with this method.


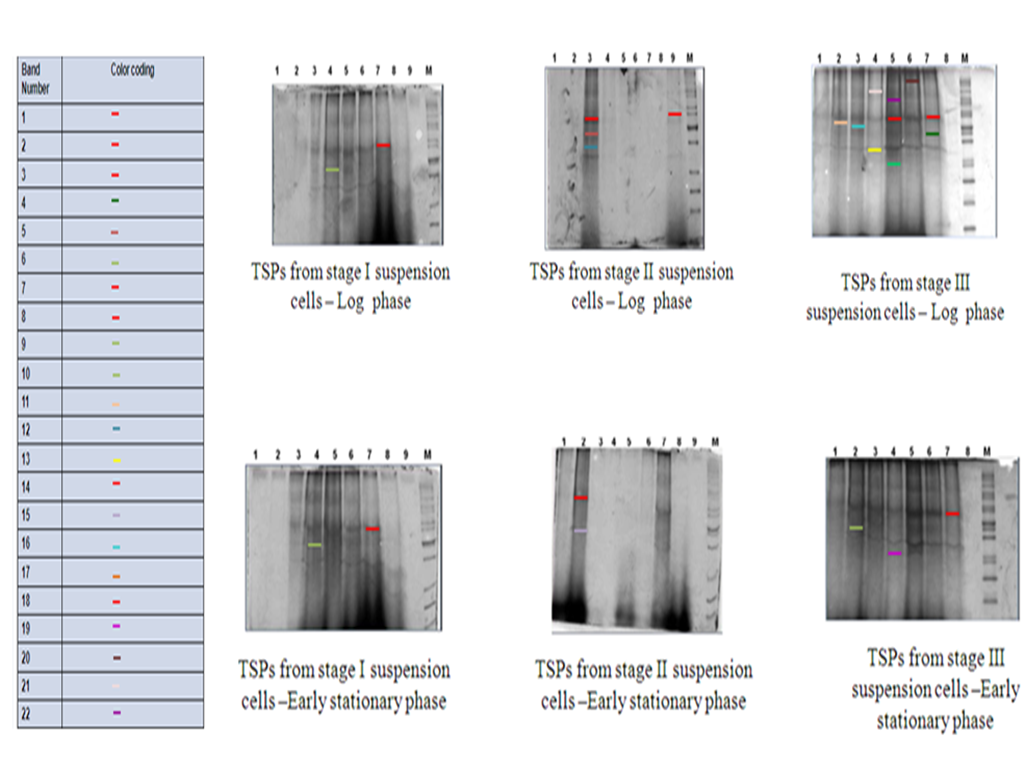


**Supplementary Figure 3.** SDS PAGE of total soluble proteins from suspensions I-III representing different ripening stages of the explants and separated by liquid phase IEF in a Microrotofor cell. TSP’s refers to total soluble proteins. Left to Right – Lanes – 1-9 – Fractions harvested from the microrotofor cell separated according to their pI. Lane 1 being most acidic and Lane 9 being highly basic. Color coding scheme has been used to denote bands picked from the gels for MS analysis.
