# Supplementary material for: Proteomic Analysis of Grape Berry Cell Cultures Reveals that Developmentally Regulated Ripening Related Processes Can Be Studied Using Cultured Cells
Source: PLoS One. 2011 Feb 17;6(2):e14708. doi: 10.1371/journal.pone.0014708 (PMC3040747; doi:10.1371/journal.pone.0014708)
Supplement: Table S1 — MALDI-TOF identification of proteins after band matching to compare their relative expression profiles. (0.08 MB DOC) [file pone.0014708.s002.doc]

| **Band No** | **Protein ID** | **Accession Number** | **MOWSE**  **Score** | **Peptides matched against the total peptides** | **Sequence Coverage** | **Experimental**  **Mass (kilo Daltons)** | **Theoretical Mass / pI** | **Error** | **Expression profile (suspensions I-III)** |
| --- | --- | --- | --- | --- | --- | --- | --- | --- | --- |
| **1** | **Class I β-1,3-glucanase (*Vitis vinifera*)** | gi/82547239 | 101 | 10/ 49 | 36 % | 42 | 39353/ 9.31 | 4e - 05 |  |
| **2** | **Class I β-1,3-glucanase (*V. vinifera*)** | gi/82547239 | 111 | 12 /60 | 47 % | 42 | 39353/ 9.31 | 4e – 06 |  |
| **3** | **Class I β-1,3-glucanase (*V. vinifera*)** | gi/82547239 | 155 | 14/49 | 57 % | 42 | 39353/ 9.31 | 1.6 e – 10 |  |
| **4** | **Predicted hypothetical protein (*V. vinifera*)** | gi/225456483 | 73 | 13 /128 | 38 % | 41 | 35559 / 8.16 | 0.0025 |  |
| **5** | **Unnamed protein product (*V. vinifera*)** | gi/157353008 | 70 | 12 / 106 | 45 % | 37 | 32893 / 8.64 | 0.0053 |  |
| **6** | **Predicted hypothetical protein (*V. vinifera)*** | gi/242042425 | 77 | 8/ 69 | 48 % | 35 | 29317 / 9.13 | 0.29 |  |
| **7** | **Class I β-1,3-glucanase (*V. vinifera*)** | gi/225431926 | 115 | 13 /69 | 48 % | 42 | 39353/ 9.31 | 8e – 07 |  |
| **8** | **Class I β-1,3-glucanase (*V. vinifera*)** | gi/1658001 | 88 | 8 /31 | 51 % | 42 | 39353/ 9.31 | 0.00088 |  |
| **9** | **Predicted hypothetical protein (*V. vinifera)*** | gi/242042425 | 77 | 8/69 | 48 % | 35 | 29317 / 9.13 | 0.29 |  |
| **10** | **Predicted hypothetical protein (*V. vinifera)*** | gi/242042425 | 82 | 12/ 44 | 42 % | 35 | 29317 / 9.13 | 0.0061 |  |
| **11** | **Predicted hypothetical protein (*V. vinifera)*** | gi/147769004 | 82 | 8 / 49 | 57 % | 42 | 39353 / 9.31 | 0.051 |  |
| **12** | **Unnamed protein product (*V. vinifera*** | gi/157335950 | 80 | 12 /106 | 45 % | 40 | 32893 / 8.64 | 0.0053 |  |
| **13** | **Ankyrin protein kinase (*Zea mays)*** | gi/226495939 | 73 | 12/18 | 38% | 38 | 35559/ 8.16 | 0.0025 |  |
| **14** | **Class I β-1,3-glucanase (*V. vinifera*)** | gi/82547239 | 81 | 10 / 22 | 36 % | 42 | 39353/ 9.31 | 0.0042 |  |
| **15** | **Ribosomal protein S2** | gi/108796848 | 90 | 8 / 10 | 31% | 33 | 26431/ 9.15 | 0.00025 |  |
| **16** | **Ferredoxin NADPH Oxidoreductase (*Triticum aestivum*)** | Q8RVZ9_WHEAT | 91 | 15/ 85 | 39 % | 40 | 38782 / 8.29 | 0.0001 |  |
| **17** | **Ras related GTP binding protein** | gi/89257659 | 160 | 16 / 21 | 51 % | 29 | 26374/ 8.80 | 2.5e-11 |  |
| **18** | **Class I β-1,3-glucanase (*V. vinifera*)** | gi/82547239 | 101 | 10 / 49 | 36 % | 42 | 39353/ 9.31 | 4e -05 |  |
| **19** | **Lys M domain containing receptor like kinase** | gi/163257407 | 81 | 7/59 | 72 % | 14 | 11499 / 9.01 | 0.0043 |  |
| **20** | **TIR NBS TIR type disease resistance protein** | Q19PM3_POPTR | 70 | 10 / 29 | 12 % | 142 | 149778/ 6.27 | 0.014 |  |
| **21** | **Malate dehydrogenase**  **(*V. vinifera)*** | gi/1561774 | 130 | 15/ 19 | 28 % | 72 | 69942/ 5.98 | 2.5e-08 |  |
| **22** | **Glutamate dehydrogenase**  **(*V. vinifera)*** | gi/806595 | 133 | 13/ 17 | 35 % | 51 | 44518 / 6.28 | 1.2e-08 |  |

**Table S1:** MALDI-TOF identification of proteins after band matching to compare their relative expression profiles.

The data was derived from the late log phases of suspension cultures I-III (originating from green, véraison and ripe berry explants respectively). 5 µl Page ruler unstained protein ladder (SM 0661, Fermentas) was run along with the vacuum harvested proteins to determine the experimental molecular weight. The error in the table indicates the mass tolerance setting. A significant match can be obtained when the mass tolerance approaches 1%. With a larger number of peptides, a mass tolerance of better than 0.2% is required for a significant match. If the number of peptides generated is less, the match is significant only when the mass tolerance is close to 1%. The densitometry values for each of the individually identified proteins are provided in Table S2.
