# Supplementary material for: Proteomic Analysis of Grape Berry Cell Cultures Reveals that Developmentally Regulated Ripening Related Processes Can Be Studied Using Cultured Cells
Source: PLoS One. 2011 Feb 17;6(2):e14708. doi: 10.1371/journal.pone.0014708 (PMC3040747; doi:10.1371/journal.pone.0014708)
Supplement: Table S3 — Proteomic studies on grape berry ripening and expression profile comparison of proteins identified in our study with the published literature. (0.07 MB DOC) [file pone.0014708.s004.doc]

**Table S3.** Summary of published proteomic studies on grape berry ripening and comparison of protein expression profiles obtained in our study with the published literature

| **Reference** | **Title of the article** | | **Stages of grape berry ripening studied** | | ***Vitis vinifera* cv** | **Technology used for profiling** | **Summary of findings** | | |
| --- | --- | --- | --- | --- | --- | --- | --- | --- | --- |
| **Deluc et al. 2007. BMC Genomics 8, 429.** | Transcriptomic and metabolite analyses of Cabernet Sauvignon during grape berry development | | Seven stages of berry development from small pea size berries, through véraison, to mature berries | | Cabernet Sauvignon | GeneChip® Vitis oligonucleotide microarray ver. 1.0 | 28% of transcripts, 4,151 Unigenes, had pronounced (≥2 fold) differences in mRNA expression.  mRNA expression patterns of transcription factors, abscisic acid (ABA) biosynthesis, and calcium signaling genes identified.  Candidate genes likely to participate in the progression of key developmental events such as véraison and potential candidate genes associated with such processes as auxin partitioning within berry cells, aroma compound production. | | |
| **Zhang et al. 2008**  **Journal of Experimental Botany 59: 2979-2990.** | Grape berry plasma membrane proteome analysis and its differential expression during ripening | | Berries were harvested 50 days after flowering , at véraison (75 days after flowering) and at ripening (95 days after flowering) | | Cabernet Sauvignon | Plasma membrane from different stages and 2DE and MALDI-TOF/MS | Twelve proteins showed significant differences in relative abundance. Ubiquitin proteolysis was found to dramatically decrease at véraison. Functional classification of the plasma membrane proteins reveals that most of the proteins were metabolic and cellular and the relative abundance was more dynamic than progressive. | | |
| **Deytieux C. 2007. Journal of experimental Botany 58: 1851-1862.** | Proteome analysis of grape skins during ripening | | Grape clusters were collected at different stages of ripening. 46 days after anhtesis corresponding to onset of skin color change or véraison , 55 days after anthesis where the berries were red and 94 days after anthesis (full maturity) | | Cabernet Sauvignon | Skins from different stages were used for 2DE and MALDI-TOF/MS | Proteins involved photosynthesis, carbohydrate metabolism and stress response was over expressed at véraison and anthocyanin. Glucanases were found to increase in abundance at ripening | | |
| **Lucker et al. 2009. BMC Genomics 10: 50.** | Generation of a predicted protein database and application of iTRAQ analyses in grape (*Vitis vinifera* cv. Cabernet Sauvignon) berries at ripening initiation | | Grapes were collected at 50 % ripening initiation based on a turning pink color phenotype. Samples represented four developmental stages. Green, pink turning stage, fully turned and purple. | | Cabernet Sauvignon | Total proteins from exocarp and mesocarp were used for iTRAQ. | The data showed that key components of ABA and BR biosynthesis were found in abundance at ripening. Influx of proteins related to accumulation of sugars and anthocyanin biosyntheis was also established. | | |
| **Gribaldi et al. 2007. Proteomics 7: 3154-3170.** | Analysis of protein changes during grape berry ripening by 2DE and MALDI TOF | | Berries were collected at 10-day intervals starting from one month after flowering until complete ripening for a total of seven sampling dates | | Nebbiola Lampia | Total soluble proteins from seven different stages were used for 2DE and MALDI-TOF/MS | 119 proteins were found to be differentially expressed during berry development. Majority of the proteins belonged to metabolism, energy and protein synthesis. General decrease of glycolysis was noticed during ripening. Oxidaive stress decreases during ripening and extensive cytoskeleton rearrangement takes place at ripening. | | |
| **Sarry et al. 2004. Proteomics 4: 201-215.** | Grape berry biochemistry revisited upon proteomic analysis of the mesocarp | | Berries harvested at ripe stage when the hexose concentration reached 1.1 M. | | Ugni blanc  Gamay noir  Dattier de Beyrouth  Muscat d Alexandrie | Total soluble proteins from berries were used for 2DE and MALDI-TOF/MS | Results strongly support apoplastic sugar loading during ripening. The study also identified novel ripening- induced proteins. | | |
| **Ferri et al. 2009. Proteomics 9: 610-624.** | Chitosan treatment induces changes of protein expression profile and stilbene distribution in *Vitis vinifera* cell suspensions | | Cell suspensions were treated with 50 μg/ml chitosan | | Callus established from leaf petioles of *Vitis vinifera* cv. Barbera | Total soluble proteins from the elicited cells were used for 2DE and MALDI-TOF/MS | Chitosan induces *V. vinifera* cell suspension cultures to increase defense mechanisms related to intracellular content of a large spectrum of anti-oxidants | | |
| **Pilati et al. 2007.**  **BMC Genomics 8: 428** | Genome wide transcriptional analysis of grapevine berry ripening reveals a set of genes similarly modulated during three seasons and the occurrence of an oxidative burst at véraison | | Grape clusters were collected from floweing to over ripening. | | Pinot Noir | Total RNA was used for microarray using the Affymetrix Genechip Vitis arrays. | 19% of the core gene set was characterized by genes involved in regulatory processes, such as transcription factors and transcription factors related to hormonal metabolism and signal transduction. In addition, an oxidative burst previously not detected in grapevine, characterized but rapid accumulation of hydrogen peroxide starting from véraison and modulated by many ROS scavenging enzymes was observed. | | |
| **Waters et al. 2006. Plant Science 171: 132-138** | The ripening wine grape berry skin transcriptome | | Samples collected 13 weeks post flowering. | | Shiraz,  Cabernet Sauvignon,  Pinot Noir,  Chardonnay,  Riesling,  Sauvignon Blanc,  Semillon | Berry skin and flesh were separated and used for microarray analysis | Only a small proportion of the genes within the berry skin showed a three-fold or greater difference in expression level after ripening commenced. Most of the differences appear to arise from environmental signals rather than genome differences. | | |
| **Grimplet et al. 2009. Proteomics 9: 2503-2528.** | Proteomic and selected metabolite analysis of grape berry tissues under well – watered and water-deficit stress conditions | | Berries were harvested at ripening stage. | | Cabernet Sauvignon | Soluble proteins were used for 2DE and MALDI-TOF/MS | Water deficit stress altered the abundance of approximately 7% of pericarp proteins,but had little effect on seed protein expression. | | |
| **EXPRESSION PATTERNS OF PROTEINS IDENTIFIED IN OUR STUDY AS DESCRIBED IN LITERATURE** | | | | | | | | | |
|  | **Class I β-1,3-glucanase (*Vitis vinifera*)** | **Ankyrin protein kinase (*Zea mays)*** | **Ribosomal protein S2** | **Ferredoxin NADPH Oxidoreductase (*Triticum aestivum*)** | **Ras related GTP-binding protein** | **Lys M domain containing receptor like kinase** | **TIR NBS TIR type disease resistance protein** | **Malate**  **dehydrogenase** | **Glutamate dehydrogenase** |
| **Expression profile in the current study** | It recorded low expression at stage one, increased levels at stage two with a slight decrease during ripening. | It had low expression at stage one, increased at stage two and showed a modest decrease in stage three | It was found to decrease in concentration in stage two and increases in stage three. | This protein records a constant lower expression at green and véraison stages with a sharp increase at the ripening stage. | The expression is higher at stage one followed by a decrease at stage two and a slight increase later in stage three. | The expression of this protein dropped at véraison but increased again during ripening. | This protein had lower activities at both stage one and two with a sharp increase in stage three. | The MDH expression is low at the stage one, increases at stage two and is slightly higher in stage three. | The levels of glutamate dehydrogenase (GDH) identified in our study were very high at stage one, decreased sharply at in stage two and remained constant thereafter. |
| **Deluc et al. 2007.** | Expressed across all stages of berry development. | Kinase and related proteins show a steady state increase at all stages of berry development. | ___ | Steady state decline at all stages of berry development. | ___ | Steady state increase at all stages of berry development. | ___ | Increases at ripening. | Transient peak decrease at véraison. |
| **Zhang et al. 2008** | ___ | ___ | Slight reduction at véraison . | ___ | They were abundant in pre véraison berries and increased post véraison . | ___ | ___ | ___ | ___ |
| **Deytieux et al. 2007.** | Proteins over-expressed at maturity and ripening. | ___ | ___ | Similar group of proteins are 73 times more abundant at ripening. | ___ | ___ | ___ | ___ | ___ |
| **Lucker et al. 2009.** | Several protein isoforms of glucanase significantly increased along ripening initiation. | It increases gradually at all stages. | No significant changes. | Shows no significant changes and is found in the mesocarp. | No significant changes. | Similar group of receptor kinases were found in mesocarp and their pattern and did not change significantly. | ___ | Increases gradually and found in the mesocarp. | No significant changes. |
| **Gribaldi et al. 2007.** | Activity remains undetected during ripening. | Similar group of kinases show gradual increase at ripening. | Decreases after véraison . | High activites at unripe stage and decreases later. | Decreases sharply at véraison . | Gradually increases at all stages. | ___ | Remains constant. | Remains constant with a slight decrease during ripening. |
| **Sarry et al. 2004.** | Higher expression at ripening. | ___ | ___ | No significant changes at ripening. | No significant changes at ripening. | No significant changes at ripening. | ___ | Both cytosolic and mitochondrial proteins are abundant at ripe stage. | ___ |
| **Ferri et al. 2009.** | Induced in response to chitosan treatment. | ___ | 2.9-fold increase after chitosan elicitation. | 2.5-fold increase after chitosan elicitation. | ___ | ___ | ___ | 2-fold increase after chitosan elicitation. | ___ |
| **Pilati et al. 2007.** | Induced strongly on response to ethylene. | No significant changes. | No significant changes. | ___ | ___ | No significant changes. | ___ | Strongly induced at véraison . | ___ |
| **Waters et al. 2006.** | No significant changes. | Upregulated at ripening. | ___ | ___ | ___ | Upregulated at ripening. | ___ | ___ | ___ |
| **Grimplet et al. 2009.** | 0.43-fold increase due to water stress in the pulp. | ___ | 2.36-fold decrease due to water stress. | ___ | ___ | 0.17-fold increase in the skin due to water stress. | ___ | 2.07-fold decrease due to water stress. | 0.29-fold increase in the pulp due to water stress. |
